# Endoscopic ultrasound‐guided tissue acquisition allows a reliable proliferation assessment of small (≤20 mm) pancreatic neuroendocrine tumors

**DOI:** 10.1002/ags3.12871

**Published:** 2024-10-09

**Authors:** Yoshihide Nanno, Hirochika Toyama, Kazuyuki Nagai, Dongha Lee, Yuichiro Uchida, Jun Ishida, Takeshi Takahara, Ippei Matsumoto, Etsuro Hatano, Takumi Fukumoto

**Affiliations:** ^1^ Division of Hepato‐Biliary‐Pancreatic Surgery, Department of Surgery Kobe University Graduate School of Medicine Kobe Hyogo Japan; ^2^ Division of Hepato‐Biliary‐Pancreatic Surgery and Transplantation, Department of Surgery Graduate School of Medicine, Kyoto University Kyoto Kansai Japan; ^3^ Division of Hepato‐Biliary‐Pancreatic Surgery, Department of Surgery Kindai University Faculty of Medicine Osaka Kansai Japan; ^4^ Department of Surgery Fujita Health University Toyoake Aichi Japan

**Keywords:** endoscopic ultrasonography, pancreatic neuroendocrine tumor, preoperative assessment, tumor grading, tumor size

## Abstract

**Aim:**

Evidence regarding the reliability of endoscopic ultrasound‐guided tissue acquisition (EUS‐TA) for assessing histological proliferation and WHO grading of small (≤20 mm) pancreatic neuroendocrine tumors (PanNETs) is limited.

**Methods:**

In this multicenter retrospective study, we analyzed data from 122 patients with small PanNETs who underwent EUS‐TA followed by surgical resection between 2006 and 2022. We compared the histopathological proliferation assessment and WHO grading between preoperative EUS‐TA and surgical definitive specimens.

**Results:**

Among the 122 patients with small PanNETs (80% with surgical definitive WHO grade G1 and 20% with G2), EUS‐TA histology identified neuroendocrine tumors in 101 (83%) patients and provided WHO grading in 85 (70%) patients. Histopathological WHO grading for EUS‐TA was concordant with surgical definitive grading in 86% (73/85) of cases, overstaged in 4% (3/85), and understaged in 11% (9/85). Moderate, severe, and fatal adverse events associated with EUS‐TA, as classified by the lexicon, were not reported in this cohort.

**Conclusion:**

EUS‐TA is a reliable method for assessing histopathological proliferation and WHO grading of small PanNETs. However, grading discordance may occur, and a risk–benefit evaluation on a per‐patient basis is recommended.

## Graphical abstract

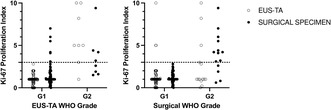

## 1 INTRODUCTION

Pancreatic neuroendocrine tumors (PanNETs) are rare pancreatic neoplasms, accounting for ~5% of all pancreatic tumors.^1^ The incidence of newly diagnosed nonfunctioning PanNETs has been steadily rising, especially in small (≤20 mm) subsets, likely due to advancements in imaging modalities such as computed tomography (CT), magnetic resonance imaging (MRI), and endoscopic ultrasonography (EUS).^2^, ^3^ Most nonfunctioning PanNETs ≤2 cm are generally considered benign, with a low likelihood of progression^1^; however, the natural history and long‐term behavior of these tumors remain inadequately understood. Guidelines generally favor observation for asymptomatic small PanNETs but accept resection for those with symptoms, suspicious radiologic or histologic findings, or based on patient preference.^4^, ^5^, ^6^, ^7^ The ambiguity of guideline recommendations imposes challenges for both physicians and patients in determining the optimal management strategy. Studies have shown that 70%–80% of patients with small nonfunctioning PanNETs undergo resection, despite the fact that the surgical intervention is not necessarily recommended.^8^, ^9^

When considering resect versus observation, several clinicopathological factors have been identified as correlates of poor prognosis in PanNETs.^10^, ^11^, ^12^, ^13^ Nevertheless, reliable preoperative assessment remains challenging.^14^, ^15^ Histological proliferation metrics, such as the Ki‐67 proliferation index and mitotic counts, along with WHO grading, on surgically definitive specimens are established prognostic factors of PanNETs available after surgery.^16^ Although preoperative histological proliferation assessment is also available before surgery via EUS‐guided tissue acquisition (EUS‐TA), the feasibility of histological proliferation assessment and WHO grading by EUS‐TA has not been established and EUS‐TA proliferation assessment is not yet included in current guidelines.^4^, ^5^, ^6^, ^7^ This is partly because EUS‐TA may not always provide an adequate quantity of number cells or tissue to meet guideline standards: The WHO guideline recommends examining a minimum of 50 high‐power fields for the mitotic count and a minimum of 500 cells counted in the hotspot area for the Ki‐67 proliferation index determination on surgical specimens.^17^ While a few studies have evaluated the utility of EUS‐TA for small (≤2 cm) PanNETs, they are limited by small sample size (≤28) and conflicting results, with insufficient evidence available on this topic.^18^, ^19^, ^20^ Therefore, we conducted this study to compare preoperative histological proliferative assessment and WHO grading by EUS‐TA with surgically definitive specimens in small (≤20 mm) PanNETs across four tertiary high‐volume referral centers. We aimed to evaluate whether EUS‐TA proliferation assessment could serve as a reliable tool for guiding the management of small PanNETs.

## 2 METHODS

### 2.1 Case selection

**FIGURE 1:**
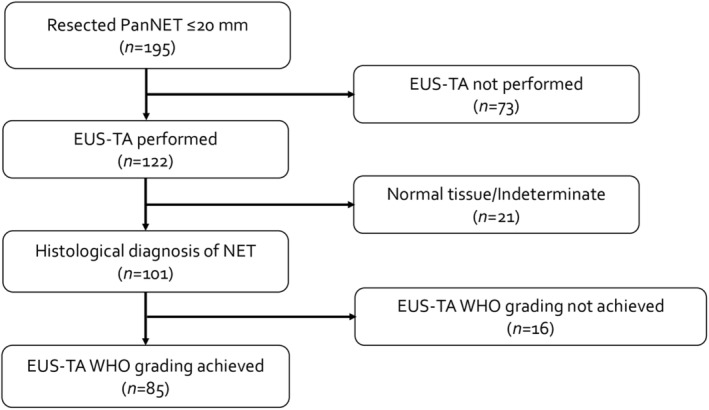
Flow diagram of the study.

The protocol for this research project was approved by the Ethics Committee of Kobe University Graduate School of Medicine (Approval No. B220249) and it conforms to the provisions of the Declaration of Helsinki. We reviewed clinicopathological data and oncological outcomes for 195 consecutive patients with surgically resected small (≤20 mm) PanNETs from four tertiary referral centers: Kyoto University Hospital, Kobe University Hospital, Kindai University Hospital, and Fujita Health University Hospital, between April 2006 and December 2022. Preoperative histological assessment by EUS‐TA was performed in 122 patients, all of whom were included in this study (Figure 1). Patients with incidentally identified PanNETs found in resected specimens for other conditions were excluded. Tumor sizes were measured based on histology slides. The largest tumor was assessed for patients with multiple tumors.

### 2.2 Histological diagnosis of PanNET and proliferation assessment

A preoperative EUS‐TA study was performed by expert endosonographers in each institution. The choice of needle type (fine‐needle aspiration [FNA] or fine‐needle biopsy [FNB]) and needle size were determined by institutional policy or the discretion of the endosonographers. Collected material was (a) processed by direct smears or cell‐block and fixed in alcohol for cytology and/or was (b) processed as a standard biopsy specimen by fixing in formalin for histology. The diagnosis of PanNET was based on morphological features, and tumor proliferation assessments were conducted according to Ki‐67 indices and mitotic counts as outlined in the World Health Organization 2017 classification.^17^ Adverse events related to EUS‐TA were classified according to the American Society for Gastrointestinal Endoscopy (ASGE) lexicon.^21^

### 2.3 Definitions of other clinical features of PanNET

Radiological features of PanNET were assessed referring to previous reports.^10^, ^11^ Tumor enhancement characteristics were evaluated using multiphasic contrast‐enhanced CT scans, and tumor attenuation was compared visually with the adjacent pancreas in the late arterial phase and categorized according to their enhancement characteristics.^10^ Main pancreatic duct (MPD) involvement was defined as stenosis of the MPD at the tumor mass, accompanied by upstream dilatation of the MPD that was more than double the diameter of the proximal MPD.^11^

### 2.4 Statistical analysis

Patient characteristics are reported as counts (and percentage) for categorical variables and compared using the Fisher's exact test, and reported as medians (and interquartile ranges [IQR]) for continuous variables and compared using the Mann–Whitney's *U* test. Multivariate logistic regression models were employed to predict successful histological diagnosis of NET and histological proliferative assessment, including all predictors with *p* < 0.05 from univariate regression analyses. Correlation coefficients were calculated using the nonparametric Kendall rank correlation test and results expressed in terms of tau coefficients. All analyses were performed with JMP Pro 16.0 for Macintosh (SAS Institute, Inc., Cary, NC, USA).

## 3 RESULTS

### 3.1 Demographic characteristics

A total of 122 patients with a median age of 62 y (IQR 52–68) at the time of surgery were included in the analysis (Table 1). This cohort included 11 patients with multiple lesions, but none of the cases involved multiple EUS‐TA procedures for different lesions. The study focused on the performance of EUS‐TA and included both nonfunctioning (*n* = 94, 77%) and functioning (*n* = 28, 23%) PanNETs. Two patients (2%) had a history of multiple endocrine neoplasia type 1, and one patient (1%) had von Hippel–Lindau disease. The median tumor diameter was 11 mm (9–15 mm), and the tumor location included the pancreatic head‐diffuse in 42 patients (34%) and body‐tail in 80 patients (66%). Surgical procedures performed included pancreatoduodenectomy/total pancreatectomy (*n* = 28, 23%), distal pancreatectomy (*n* = 52, 43%), or middle pancreatectomy/enucleation (*n* = 42, 34%). The definitive WHO histological classification obtained on surgical specimen was G1 in 97 patients (80%) and G2 in 25 patients (20%).

**TABLE 1:** Patient characteristics.

Variables	All
Number of patients	122
Age, y	62 (52–68)
Male sex	54 (44%)
Tumor size, mm	11 (9–15)
Number of pancreatic masses	
Single	111 (91%)
Multiple	11 (9%)
Tumor location	
Head‐diffuse	42 (34%)
Body‐tail	80 (66%)
Tumor type	
Nonfunctioning	94 (77%)
Insulinoma	25 (20%)
Gastrinoma	2 (2%)
Glucagonoma	1 (1%)
CT enhancement type	
Hyper	89 (73%)
Hetero/hypo	29 (24%)
Not assessed	4 (3%)
MPD involvement	
Negative	113 (93%)
Positive	9 (7%)
Type of surgery performed	
PD/TP	28 (23%)
DP	52 (43%)
MP/EN	42 (34%)
Histological diagnosis	
NET G1	97 (80%)
NET G2	25 (20%)
Nodal metastasis	
Negative	114 (93%)
Positive	8 (7%)
Distant metastasis	
Negative	120 (98%)
Positive	2 (2%)
Ki‐67 index, %	1 (1–2)
Mitotic count,^a^ per 10 HPF	1 (0–1)
Lymphatic invasion^b^	
Negative	89 (96%)
Positive	4 (4%)
Venous invasion^b^	
Negative	85 (91%)
Positive	8 (9%)
Perineural invasion^c^	
Negative	87 (95%)
Positive	5 (5%)

*Note*: Values are expressed as median (interquartile range; IQR) or *n* (%).

^a^ Missing in four patients.

^b^ Missing in 29 patients.

^c^ Missing in 30 patients.

### 3.2 Diagnosis of PanNET by EUS‐TA

Table 2 summarizes the details of the EUS‐TA procedures. The largest needle size used for EUS‐TA was 22‐G in 74 patients (61%) and 25‐G in 18 patients (15%). The median number of punctures was 3 (range, 1–8). FNA needles were used in 60 patients (49%) and FNB needles in 56 patients (46%), with 16 patients undergoing both FNA and FNB needles. The needle type was unknown in 22 patients (18%). Although three mild adverse events related to EUS‐TA were reported according to the ASGE lexicon (one case each of pancreatitis, fever, and nausea/vomiting), no moderate, severe, or fatal events were observed.

**TABLE 2:** Details of the EUS‐TA trials.

Variables	*n* = 122
Number of EUS‐TA trials, *n*	
Mean	2.9
Median	3
Min, Max	1, 8
Needle size, largest, *n* (%)	
19G	4 (3%)
20G	1 (1%)
21G	4 (3%)
22G	74 (61%)
25G	18 (15%)
Not reported	21 (17%)
Needle type, *n* (%)	
FNA needles only	44 (36%)
FNB needles only	40 (33%)
Both types	16 (13%)
Not reported	22 (18%)

Among the 122 patients who underwent preoperative EUS‐TA, 101 patients (83%) received a pathological diagnosis of PanNET. Pathological diagnosis of PanNET was less likely to be achieved among patients with hypo‐attenuation patterns on enhanced CT (*p* = 0.017) and those with MPD involvement (*p* = 0.047). No significant difference was found regarding tumor size (*p* = 0.755), tumor location (*p* = 0.258), or other patient and tumor characteristics (Tables S1).

### 3.3 Histological proliferation assessment and WHO grading by EUS‐TA

**FIGURE 2:**
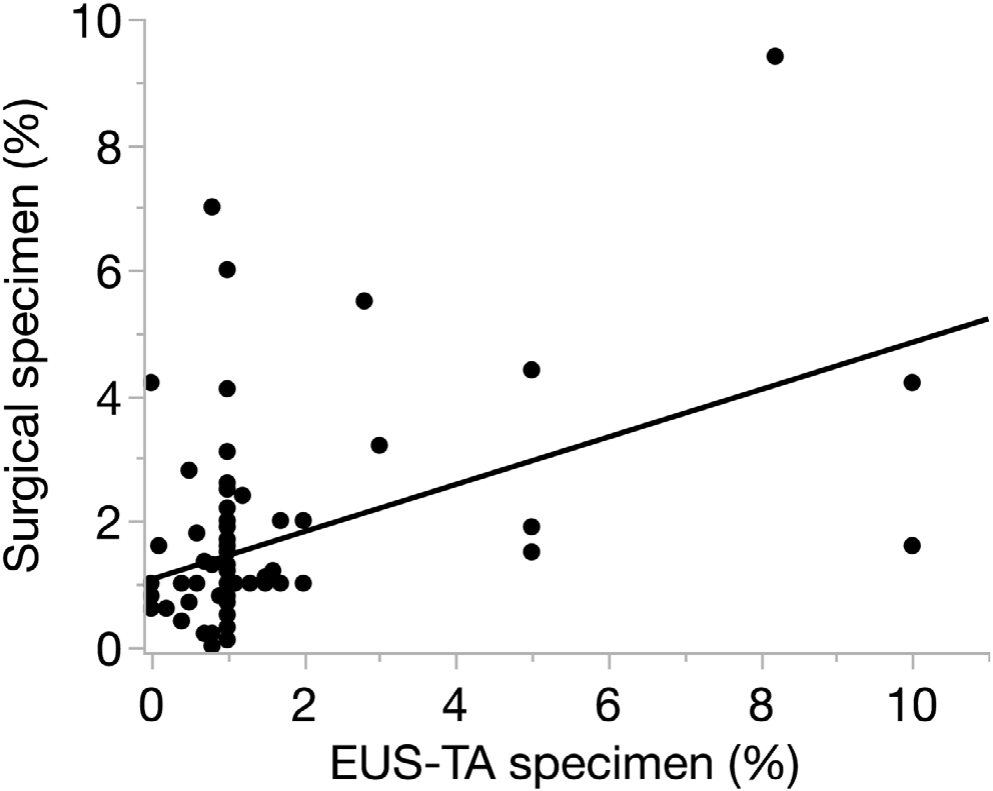
Correlation of Ki‐67 proliferation index between EUS‐TA and surgical specimens.

**FIGURE 3:**
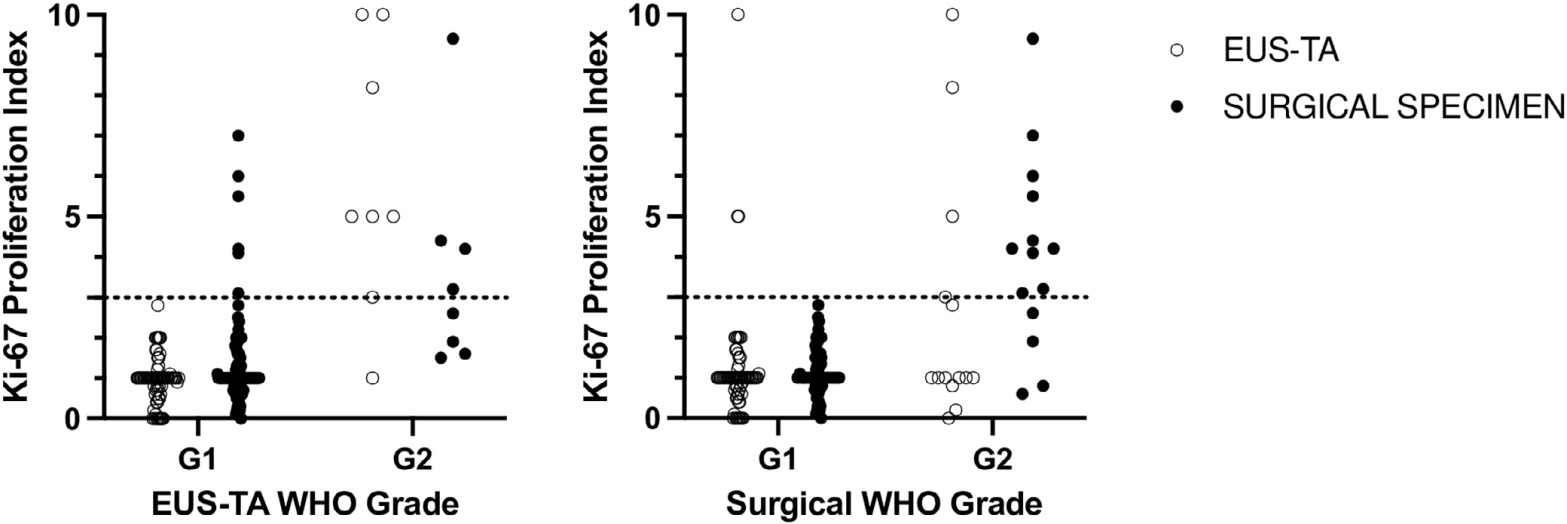
Distribution of Ki‐67 proliferation indices in EUS‐TA versus surgical histological assessment.

Of the 101 patients diagnosed with PanNET, 85 patients (84%) had their WHO grading assessment (Figure 1). There was a fair correlation of the Ki‐67 proliferation index between EUS‐TA and surgical specimens (*τ* = 0.282, *p* < 0.001; Figure 2). WHO grading on EUS‐TA was concordant with surgical definitive grading in 86% (73 of 85 patients) when using the 3% cutoff and 80% (64 of 85 patients) when using the 5% cutoff (Table 3). With the 3% cutoff, nine patients (11%) had an upgrade in WHO grading (G1 by EUS‐TA to G2 by surgical specimen), while three patients (4%) had a downgrade (G2 by EUS‐TA to G1 by surgical specimen). Grading agreement for G1 tumors was 88% (68 of 77 patients) and 63% (five of eight patients) for G2 tumors (Table 3). Among the 16 patients for whom WHO grading could not be assessed by EUS‐TA, five (31%) were classified as G2 on surgical specimen (Table 3). Among the 77 G1 PanNETs assessed by EUS‐TA, six (8%) had Ki‐67 proliferation indices ≥3% on surgical histology (Figure 3). Contrarily, among the 14 G2 PanNETs diagnosed surgically, ten (71%) and four (29%) had Ki‐67 proliferation indices <3% on EUS‐TA and surgical histology, respectively (Figure 3).

**TABLE 3:** Comparisons of WHO gradings between EUS‐TA and surgical specimens.

Ki‐67 cutoff 3%	EUS‐TA specimen		
	NET G1 (*n* = 77)	NET G2 (*n* = 8)	NET Gx (*n* = 16)
Surgical specimen			
NET G1	68 (88%)	3 (37%)	11 (69%)
NET G2	9 (12%)	5 (63%)	5 (31%)

Ki‐67 cutoff 5%	EUS‐TA specimen		
	NET G1 (*n* = 78)	NET G2 (*n* = 7)	NET Gx (*n* = 16)
Surgical specimen			
NET G1	64 (82%)	3 (43%)	11 (69%)
NET G2	14 (18%)	4 (57%)	5 (31%)

The concordance rate of EUS‐TA WHO grading with surgical specimens was consistent across different tumor sizes in 5‐mm increments, with no significant differences among the groups, regardless of whether the 3% (*p* = 0.459) or 5% (*p* = 0.420) cutoff was used (Table 4).

**TABLE 4:** Summary of concordance rates between EUS‐TA WHO grading and surgical definitive grading, stratified by tumor size.

Tumor size (mm)	Accuracy for 3% cutoff	Accuracy for 5% cutoff
>15 and ≤20	93% (14/15)	80% (12/15)
>10 and ≤15	77% (24/31)	71% (22/31)
>5 and ≤10	89% (31/35)	86% (30/35)
≤5	100% (4/4)	100% (4/4)

### 3.4 Factors affecting diagnostic performance of EUS‐TA

**FIGURE 4:**
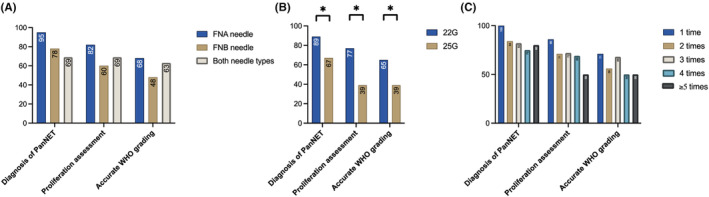
Success rates of making pathological diagnosis, histopathological proliferation assessment, and accurate WHO grading according to (A) needle types, (B) needle sizes, and (C) number of punctures.

We analyzed factors associated with the diagnostic performance of EUS‐TA, including needle type (FNA or FNB), needle size (22G or 25G), and number of punctures (Figure 4). The use of a 22G needle was significantly associated with better histopathological diagnostic accuracy compared to the 25G needle. No significant differences were found between needle types or number of punctures. Multivariate logistic regression analyses confirmed that the use of a 22G needle was an independent factor for achieving a successful histological diagnosis of NET and accurate histological proliferative assessment (Tables S1 and S2).

## 4 DISCUSSION

The management of small (≤20 mm) PanNETs poses significant challenges. Preoperative histological grading is crucial for guiding treatment decisions; however, the reliability of EUS‐TA for assessing histological proliferation and WHO grading in small PanNETs has not been well established. In this multi‐institutional study of surgically resected small PanNETs, we evaluated the reliability of EUS‐TA for diagnosing PanNETs and assessing tumor proliferation compared to definitive surgical specimens. Our findings indicate that EUS‐TA achieved a diagnostic accuracy of 83% and a WHO grading concordance rate of 86%, suggesting that EUS‐TA can be a valuable preoperative tool.

The 86% concordance rate of WHO grading between EUS‐TA and surgical specimens in our study is consistent with the previous study, which reported an estimated concordance rate of 80% across various tumor sizes (95% CI: 76%–85%).^22^ These results support an observation strategy if the tumor is graded as G1 by EUS‐TA. In contrast, a recent study of 401 patients, including only small (≤2 cm) PanNETs, reported a lower overall concordance rate of 77%.^23^ This discrepancy may be attributed to the inclusion of older cases (study period: 1996–2019). Partelli et al reported a low concordance rate for EUS‐TA‐derived WHO grading, with three out of four patients initially graded as G2 by EUS‐TA being downgraded to G1 in final pathology, and concluded that EUS‐TA should not be used for preoperative assessment.^20^ In our study, three out of eight (38%) EUS‐TA G2 PanNETs were downgraded to G1, and 9 out of 77 (12%) EUS‐TA G1 PanNETs were upgraded to G2 in surgical pathology. These findings highlight the need to consider other patient factors, such as age, comorbidities, and patient preferences, when deciding on treatment strategies.

Discrepancy in grading between EUS‐TA and surgical specimens may be attributed to several factors. Hijioka et al reported that tumors with significant stromal fibrosis negatively impact sampling adequacy.^24^ Similarly, our study found that pathological diagnosis was less likely to be achieved in tumors with significant stromal fibrosis, which may be associated with MPD involvement (Table S1). Needle performance is another crucial factor. Our study, along with others, suggests that larger needles (22G) improve diagnostic accuracy for PanNET and provide more accurate WHO grading compared to smaller needles (25G).^25^ Additionally, large randomized controlled trials have shown that FNB needles generally outperformed FNA needles.^26^ Although our study found no significant difference between FNA and FNB needles, the inclusion of both first‐ and second‐generation FNB needles may have mitigated the difference. In contrast, our study analyzed the diagnostic accuracy in 5‐mm increments of tumor diameter and found that EUS‐TA and proliferation assessments were accurate regardless of tumor size (Table 4). Collectively, these findings suggest that enhancing needle performance in EUS‐TA, including optimizing needle types, could improve diagnostic accuracy of EUS‐TA, leading to more reliable preoperative assessments when determining treatment strategies.

Multiple imaging modalities are also currently employed in the preoperative assessment of nonfunctioning PanNETs. Multiple‐phasic contrast‐enhanced CT scan is a basic imaging modality for diagnosis and assessment of aggressiveness of the tumor, and a wide range of CT characteristics have been reported to be associated with poor prognosis.^27^ Yet, to the best of our knowledge, a comprehensive analysis of CT characteristics that focused on aggressiveness of small PanNETs has not yet been established. Functional imaging studies, such as ^18^F‐FDG‐PET/CT,^28^ ^68^Ga‐DOTANOC‐PET/CT,^29^ dual‐tracer functional imaging ^68^Ga‐DOTANOC, and ^18^F‐FDG‐PET/CT,^30^ as well as MRI,^31^ have shown prognostic value for nonfunctioning PanNETs. Nevertheless, their application to small (≤2 cm) PanNETs has not been well documented, and further studies are warranted.

This study has several limitations. First, the retrospective design did not control for EUS‐TA and histopathological proliferation assessment, but this may potentially reflect real‐world clinical practices. Second, confounding factors affecting EUS‐TA procedures may not be entirely controlled due to the retrospective nature of the study. Third, the analysis was limited to surgical cases with EUS‐TA; data on nonsurgical cases post‐EUS‐TA or surgical cases without EUS‐TA were not available, which restricted the assessment of diagnostic accuracy to a limited context. Fourth, while our study focused on concordance of proliferation indices, future prospective studies should evaluate whether EUS‐TA‐derived proliferation assessments and WHO grading contribute to survival benefits.

In conclusion, our study demonstrates that preoperative EUS‐TA is a reliable tool for assessing histopathological proliferation and WHO grading of small PanNETs. However, discrepancies in grading can occur, underscoring the need for individualized preoperative assessments based on a thorough risk–benefit evaluation for each patient. Future research should focus on prospectively evaluating the clinical utility of EUS‐TA in improving management strategies and survival benefits for patients with small PanNETs.

## AUTHOR CONTRIBUTIONS

**Yoshihide Nanno:** Conceptualization; data curation; formal analysis; methodology; project administration; writing – original draft. **Hirochika Toyama:** Conceptualization; formal analysis; supervision; validation; writing – original draft. **Kazuyuki Nagai:** Conceptualization; formal analysis; validation; writing – original draft. **Dongha Lee:** Conceptualization; data curation; formal analysis; writing – original draft. **Yuichiro Uchida:** Conceptualization; data curation; formal analysis; writing – original draft. **Jun Ishida:** Data curation; formal analysis; writing – original draft. **Takeshi Takahara:** Conceptualization; formal analysis; methodology; validation; writing – review and editing. **Ippei Matsumoto:** Conceptualization; formal analysis; investigation; supervision; validation; writing – review and editing. **Etsuro Hatano:** Conceptualization; supervision; validation; writing – review and editing. **Takumi Fukumoto:** Conceptualization; methodology; supervision; validation; writing – review and editing.

## FUNDING INFORMATION

The present article is not funded by any organization.

## CONFLICT OF INTEREST STATEMENT

The authors declare no conflict of interest.

## ETHICS STATEMENT

Approval of the research protocol by an Institutional Reviewer Board: The protocol for this research project was approved by the Ethics Committee of Kobe University Graduate School of Medicine (Approval No. B220249) and it conforms to the provisions of the Declaration of Helsinki. The opt‐out method to obtain patient consent was utilized at each institution.

Informed Consent: N/A.

Registry and the Registration No. of the study/trial: N/A.

Animal Studies: N/A.

## Supporting information


Table S1.

Table S2.

## Data Availability

The data that support the findings of this study are available from the corresponding author upon reasonable request.

## References

[1] Ricci C , Partelli S , Landoni L , Rinzivillo M , Ingaldi C , Andreasi V , et al. Sporadic non‐functioning pancreatic neuroendocrine tumours: multicentre analysis. Br J Surg. 2021;108(7):811–816. 33724300 10.1093/bjs/znaa141

[2] Dasari A , Shen C , Halperin D , Zhao B , Zhou S , Xu Y , et al. Trends in the incidence, prevalence, and survival outcomes in patients with neuroendocrine tumors in the United States. JAMA Oncol. 2017;3(10):1335–1342. 28448665 10.1001/jamaoncol.2017.0589 PMC5824320

[3] Vagefi PA , Razo O , Deshpande V , McGrath D , Lauwers GY , Thayer SP , et al. Evolving patterns in the detection and outcomes of pancreatic neuroendocrine neoplasms: the Massachusetts General Hospital experience from 1977 to 2005. Arch Surg. 2007;142(4):347–354. 17438169 10.1001/archsurg.142.4.347 PMC3979851

[4] Ito T , Masui T , Komoto I , Doi R , Osamura RY , Sakurai A , et al. JNETS clinical practice guidelines for gastroenteropancreatic neuroendocrine neoplasms: diagnosis, treatment, and follow‐up: a synopsis. J Gastroenterol. 2021;56(11):1033–1044. 34586495 10.1007/s00535-021-01827-7 PMC8531106

[5] Clark OH , Benson AB 3rd , Berlin JD , Choti MA , Doherty GM , Engstrom PF , et al. NCCN clinical practice guidelines in oncology: neuroendocrine tumors. J Natl Compr Cancer Netw. 2009;7(7):712–747. 10.6004/jnccn.2009.0050 19635226

[6] Falconi M , Eriksson B , Kaltsas G , Bartsch DK , Capdevila J , Caplin M , et al. ENETS consensus guidelines update for the Management of Patients with functional pancreatic neuroendocrine tumors and non‐functional pancreatic neuroendocrine tumors. Neuroendocrinology. 2016;103(2):153–171. 26742109 10.1159/000443171 PMC4849884

[7] Howe JR , Merchant NB , Conrad C , Keutgen XM , Hallet J , Drebin JA , et al. The north American neuroendocrine tumor society consensus paper on the surgical Management of Pancreatic Neuroendocrine Tumors. Pancreas. 2020;49(1):1–33. 31856076 10.1097/MPA.0000000000001454 PMC7029300

[8] Gratian L , Pura J , Dinan M , Roman S , Reed S , Sosa JA . Impact of extent of surgery on survival in patients with small nonfunctional pancreatic neuroendocrine tumors in the United States. Ann Surg Oncol. 2014;21(11):3515–3521. 24841347 10.1245/s10434-014-3769-4 PMC4510956

[9] Sharpe SM , In H , Winchester DJ , Talamonti MS , Baker MS . Surgical resection provides an overall survival benefit for patients with small pancreatic neuroendocrine tumors. J Gastrointest Surg. 2015;19(1):117–123. 25155459 10.1007/s11605-014-2615-0

[10] Mizumoto T , Toyama H , Terai S , Mukubou H , Yamashita H , Shirakawa S , et al. Prediction of lymph node metastasis in pancreatic neuroendocrine tumors by contrast enhancement characteristics. Pancreatology. 2017;17(6):956–961. 28964660 10.1016/j.pan.2017.08.003

[11] Nanno Y , Matsumoto I , Zen Y , Otani K , Uemura J , Toyama H , et al. Pancreatic duct involvement in well‐differentiated neuroendocrine tumors is an independent poor prognostic factor. Ann Surg Oncol. 2017;24(4):1127–1133. 27822631 10.1245/s10434-016-5663-8

[12] Nanno Y , Toyama H , Zen Y , Akita M , Ando Y , Mizumoto T , et al. Serum elastase 1 level as a risk factor for postoperative recurrence in patients with well‐differentiated pancreatic neuroendocrine neoplasms. Ann Surg Oncol. 2018;25(11):3358–3364. 30054822 10.1245/s10434-018-6675-3

[13] Nanno Y , Toyama H , Matsumoto I , Otani K , Asari S , Goto T , et al. Baseline plasma chromogranin a levels in patients with well‐differentiated neuroendocrine tumors of the pancreas: a potential predictor of postoperative recurrence. Pancreatology. 2017;17(2):291–294. 28043759 10.1016/j.pan.2016.12.012

[14] Partelli S , Gaujoux S , Boninsegna L , Cherif R , Crippa S , Couvelard A , et al. Pattern and clinical predictors of lymph node involvement in nonfunctioning pancreatic neuroendocrine tumors (NF‐PanNETs). JAMA Surg. 2013;148(10):932–939. 23986355 10.1001/jamasurg.2013.3376

[15] Hasegawa T , Yamao K , Hijioka S , Bhatia V , Mizuno N , Hara K , et al. Evaluation of Ki‐67 index in EUS‐FNA specimens for the assessment of malignancy risk in pancreatic neuroendocrine tumors. Endoscopy. 2014;46(1):32–38. 24218309 10.1055/s-0033-1344958

[16] Andreasi V , Ricci C , Partelli S , Guarneri G , Ingaldi C , Muffatti F , et al. Predictors of disease recurrence after curative surgery for nonfunctioning pancreatic neuroendocrine neoplasms (NF‐PanNENs): a systematic review and meta‐analysis. J Endocrinol Investig. 2022;45(4):705–718. 34773595 10.1007/s40618-021-01705-2

[17] Klöppel G , Couvelard A , Hruban RH , Klimstra DS , Komminoth P , Osamura RY , et al. WHO classification of tumors of endocrine organs. 4th ed. France: International Agency for Research on Cancer (IARC); 2017.

[18] Crinò SF , Ammendola S , Meneghetti A , Bernardoni L , Conti Bellocchi MC , Gabbrielli A , et al. Comparison between EUS‐guided fine‐needle aspiration cytology and EUS‐guided fine‐needle biopsy histology for the evaluation of pancreatic neuroendocrine tumors. Pancreatology. 2021;21(2):443–450. 33390343 10.1016/j.pan.2020.12.015

[19] Boutsen L , Jouret‐Mourin A , Borbath I , van Maanen A , Weynand B . Accuracy of pancreatic neuroendocrine tumour grading by endoscopic ultrasound‐guided fine needle aspiration: analysis of a large cohort and perspectives for improvement. Neuroendocrinology. 2018;106(2):158–166. 28494461 10.1159/000477213

[20] Partelli S , Mazza M , Andreasi V , Muffatti F , Crippa S , Tamburrino D , et al. Management of small asymptomatic nonfunctioning pancreatic neuroendocrine tumors: limitations to apply guidelines into real life. Surgery. 2019;166(2):157–163. 31109657 10.1016/j.surg.2019.04.003

[21] Cotton PB , Eisen GM , Aabakken L , Baron TH , Hutter MM , Jacobson BC , et al. A lexicon for endoscopic adverse events: report of an ASGE workshop. Gastrointest Endosc. 2010;71(3):446–454. 20189503 10.1016/j.gie.2009.10.027

[22] Tacelli M , Bina N , Crinò SF , Facciorusso A , Celsa C , Vanni AS , et al. Reliability of grading preoperative pancreatic neuroendocrine tumors on EUS specimens: a systematic review with meta‐analysis of aggregate and individual data. Gastrointest Endosc. 2022;96(6):898–908.e23. 35863518 10.1016/j.gie.2022.07.014

[23] Hijioka S , Yamashige D , Esaki M , Honda G , Higuchi R , Masui T , et al. Factors affecting nonfunctioning small pancreatic neuroendocrine neoplasms and proposed new treatment strategies. Clin Gastroenterol Hepatol. 2024;22(7):1416–1426.e5. 38615727 10.1016/j.cgh.2024.03.029

[24] Hijioka S , Hara K , Mizuno N , Imaoka H , Bhatia V , Mekky MA , et al. Diagnostic performance and factors influencing the accuracy of EUS‐FNA of pancreatic neuroendocrine neoplasms. J Gastroenterol. 2016;51(9):923–930. 26768605 10.1007/s00535-016-1164-6 PMC4990623

[25] Larghi A , Capurso G , Carnuccio A , Ricci R , Alfieri S , Galasso D , et al. Ki‐67 grading of nonfunctioning pancreatic neuroendocrine tumors on histologic samples obtained by EUS‐guided fine‐needle tissue acquisition: a prospective study. Gastrointest Endosc. 2012;76(3):570–577. 22898415 10.1016/j.gie.2012.04.477

[26] Kovacevic B , Toxværd A , Klausen P , Larsen MH , Grützmeier S , Detlefsen S , et al. Tissue amount and diagnostic yield of a novel franseen EUS‐FNB and a standard EUS‐FNA needle—a randomized controlled study in solid pancreatic lesions. Endosc Ultrasound. 2023;12(3):319–325. 37693112 10.1097/eus.0000000000000007 PMC10437204

[27] van der Velden DL , Staal FCR , Aalbersberg EA , Castagnoli F , Wilthagen E , Beets‐Tan RGH . Prognostic value of CT characteristics in GEP‐NET: a systematic review. Crit Rev Oncol Hematol. 2022;175:103713. 35598829 10.1016/j.critrevonc.2022.103713

[28] Binderup T , Knigge U , Johnbeck CB , Loft A , Berthelsen AK , Oturai P , et al. ^18^F‐FDG PET is superior to WHO grading as a prognostic tool in neuroendocrine neoplasms and useful in guiding PRRT: a prospective 10‐year follow‐up study. J Nucl Med. 2021;62(6):808–815. 33067340 10.2967/jnumed.120.244798 PMC8729872

[29] Ambrosini V , Campana D , Polverari G , Peterle C , Diodato S , Ricci C , et al. Prognostic value of ^68^Ga‐DOTANOC PET/CT SUVmax in patients with neuroendocrine tumors of the pancreas. J Nucl Med. 2015;56(12):1843–1848. 26405169 10.2967/jnumed.115.162719

[30] Majala S , Seppänen H , Kemppainen J , Sundström J , Schalin‐Jäntti C , Gullichsen R , et al. Prediction of the aggressiveness of non‐functional pancreatic neuroendocrine tumors based on the dual‐tracer PET/CT. EJNMMI Res. 2019;9(1):116. 31872324 10.1186/s13550-019-0585-7 PMC6928175

[31] De Robertis R , Cingarlini S , Tinazzi Martini P , Ortolani S , Butturini G , Landoni L , et al. Pancreatic neuroendocrine neoplasms: magnetic resonance imaging features according to grade and stage. World J Gastroenterol. 2017;23(2):275–285. 28127201 10.3748/wjg.v23.i2.275 PMC5236507

