# Supplementary material for: Endoscopic ultrasound‐guided tissue acquisition allows a reliable proliferation assessment of small (≤20 mm) pancreatic neuroendocrine tumors
Source: Ann Gastroenterol Surg. 2024 Oct 9;9(2):339–46. doi: 10.1002/ags3.12871 (PMC11877344; doi:10.1002/ags3.12871)
Supplement: Supplementary file 1 — Table S1. [file AGS3-9-339-s002.docx]

**Supplementary Table S1** Difference in patient characteristics between those for whom a pathological diagnosis of PanNETs was achieved or not achieved by EUS-TA

|  | Pathological diagnosis of NET | | Univariate |  | Multivariate | |
| --- | --- | --- | --- | --- | --- | --- |
| Variables | Achieved (n = 101) | Not achieved (n = 21) | *P* |  | Adjusted odds (95% CI) | *P* |
| Age, y | 60 (51-68) | 64 (58-72) | 0.078 |  |  |  |
| Male sex | 42 (42%) | 12 (57%) | 0.144 |  |  |  |
| Tumor size, mm | 11 (9-15) | 12 (8.5-16) | 0.755 |  |  |  |
| Number of pancreatic masses |  |  |  |  |  |  |
| Single | 91 (90%) | 20 (95%) | 0.688 |  |  |  |
| Multiple | 10 (10%) | 1 (5%) |  |  |  |  |
| Tumor location |  |  |  |  |  |  |
| Head-diffuse | 33 (33%) | 9 (43%) | 0.258 |  |  |  |
| Body-tail | 68 (67%) | 12 (57%) |  |  |  |  |
| Tumor type |  |  |  |  |  |  |
| Non-functioning | 75 (74%) | 19 (90%) | 0.439 |  |  |  |
| Insulinoma | 23 (23%) | 2 (10%) |  |  |  |  |
| Gastrinoma | 1 (1%) | - |  |  |  |  |
| Glucagonoma | 2 (2%) | - |  |  |  |  |
| CT enhancement type |  |  |  |  |  |  |
| Hyper | 74 (76%) | 15 (71%) | 0.780 |  |  |  |
| Hetero/Hypo | 23 (24%) | 6 (29%) |  |  |  |  |
| MPD involvement |  |  |  |  |  |  |
| Negative | 96 (95%) | 17 (81%) | *0.047* |  |  | 0.166 |
| Positive | 5 (5%) | 4 (19%) |  |  |  |  |
| Needle size, largest, n (%) |  |  |  |  |  |  |
| 22G | 66 (85%) | 8 (57%) | *0.028* |  | 5.08 (1.4-18.5) | *0.014* |
| 25G | 12 (15%) | 6 (43%) |  |  |  |  |
| Needle type, n (%) |  |  |  |  |  |  |
| FNA needles only | 53 (63%) | 7 (44%) | 0.171 |  |  |  |
| FNB needles or both types | 31 (37%) | 9 (56%) |  |  |  |  |
| Histological Diagnosis |  |  |  |  |  |  |
| NET G1 | 82 (81%) | 15 (71%) | 0.313 |  |  |  |
| NET G2 | 19 (19%) | 6 (29%) |  |  |  |  |
| Period |  |  |  |  |  |  |
| Early 2006-2016 | 40 (40%) | 12 (57%) | 0.153 |  |  |  |
| Late 2017-2022 | 61 (60%) | 9 (43%) |  |  |  |  |

Values are expressed as median (inter quartile range; IQR) or n (%). Significant *P*-values (< 0.01) are underlined. Marginally significant *P*-values (< 0.05) are in italic.
