# Supplementary material for: Endoscopic ultrasound‐guided tissue acquisition allows a reliable proliferation assessment of small (≤20 mm) pancreatic neuroendocrine tumors
Source: Ann Gastroenterol Surg. 2024 Oct 9;9(2):339–46. doi: 10.1002/ags3.12871 (PMC11877344; doi:10.1002/ags3.12871)
Supplement: Supplementary file 2 — Table S2. [file AGS3-9-339-s001.docx]

**Supplementary Table S2** Difference in patient characteristics between those for whom histological proliferation of PanNETs could or could not be assessed by EUS-TA

|  | Histological proliferation assessment | | Univariate |  | Multivariate | |
| --- | --- | --- | --- | --- | --- | --- |
| Variables | Achieved (n = 85) | Not achieved (n = 37) | *P* |  | Adjusted odds (95% CI) | *P* |
| Age, y | 59 (50-59) | 67 (58-71) | 0.005 |  | 0.95 (0.91-0.99) | *0.014* |
| Male sex | 36 (42%) | 18 (49%) | 0.556 |  |  |  |
| Tumor size, mm | 11 (9-15) | 12 (9-16) | 0.538 |  |  |  |
| Number of pancreatic masses |  |  |  |  |  |  |
| Single | 76 (89%) | 35 (95%) | 0.501 |  |  |  |
| Multiple | 9 (11%) | 2 (5%) |  |  |  |  |
| Tumor location |  |  |  |  |  |  |
| Head-diffuse | 27 (32%) | 15 (41%) | 0.409 |  |  |  |
| Body-tail | 58 (68%) | 22 (59%) |  |  |  |  |
| Tumor type |  |  |  |  |  |  |
| Non-functioning | 62 (73%) | 32 (86%) | 0.364 |  |  |  |
| Insulinoma | 20 (24%) | 5 (14%) |  |  |  |  |
| Gastrinoma | 1 (1%) | - |  |  |  |  |
| Glucagonoma | 2 (2%) | - |  |  |  |  |
| CT enhancement type |  |  |  |  |  |  |
| Hyper | 62 (77%) | 27 (73%) | 0.818 |  |  |  |
| Hetero/Hypo | 19 (23%) | 10 (27%) |  |  |  |  |
| MPD involvement |  |  |  |  |  |  |
| Negative | 81 (95%) | 32 (86%) | 0.128 |  |  |  |
| Positive | 4 (5%) | 5 (14%) |  |  |  |  |
| Needle size, largest, n (%) |  |  |  |  |  |  |
| 22G | 57 (89%) | 17 (61%) | 0.003 |  | 4.7 (1.4-16.0) | *0.015* |
| 25G | 7 (11%) | 11 (39%) |  |  |  |  |
| Needle type, n (%) |  |  |  |  |  |  |
| FNA needles or both types | 47 (66%) | 13 (45%) | 0.071 |  |  |  |
| FNB needles only | 24 (34%) | 16 (55%) |  |  |  |  |
| Histological Diagnosis |  |  |  |  |  |  |
| NET G1 | 82 (81%) | 15 (71%) | 0.313 |  |  |  |
| NET G2 | 19 (19%) | 6 (29%) |  |  |  |  |
| Period |  |  |  |  |  |  |
| Early 2006-2016 | 31 (36%) | 21 (57%) | *0.047* |  |  | 0.501 |
| Late 2017-2022 | 54 (64%) | 16 (43%) |  |  |  |  |

Values are expressed as median (inter quartile range; IQR) or n (%). Significant *P*-values (< 0.01) are underlined. Marginally significant *P*-values (< 0.05) are in italic. Odds ratio and 95% confidence interval (CI) are calculated for one unit (year) change in the variable for continuous values.
